# CT Attenuation and Cross-Sectional Area of the Pectoralis Are Associated With Clinical Characteristics in Chronic Obstructive Pulmonary Disease Patients

**DOI:** 10.3389/fphys.2022.833796

**Published:** 2022-06-03

**Authors:** Xin Qiao, Gang Hou, Jian Kang, Qiu-Yue Wang, Yan Yin

**Affiliations:** ^1^ Department of Pulmonary and Critical Care Medicine, First Affiliated Hospital of China Medical University, Shenyang, China; ^2^ Department of Pulmonary and Critical Care Medicine, Center of Respiratory Medicine, China-Japan Friendship Hospital, Beijing, China

**Keywords:** sarcopenia, COPD, computed tomography, pectoralis muscle, myosteatosis

## Abstract

**Purpose:** Muscle wasting is associated with prognosis in patients with chronic obstructive pulmonary disease (COPD). Computed tomography (CT) could serve as a method for muscle assessment due to its ability to measure both muscle quantity (e.g., cross-sectional muscle area) and muscle quality (e.g., muscle attenuation). Our study aimed to compare the differences in CT-derived pectoralis muscle cross-sectional area (PMA) and pectoralis muscle attenuation (PMT) between COPD patients and healthy controls and explore the association between PMA and PMT measurements and clinical characteristics in patients with COPD.

**Methods:** A total of 252 participants included in our analysis consisted of 80 healthy controls and 172 patients with COPD. PMA and PMT were measured from a single axial slice of the CT scan above the aortic arch. Linear regression analysis was used to determine the correlation between PMA and PMT measurements and clinical characteristics in patients with COPD. Associations were adjusted for age, sex, BMI, FEV_1_%pred, smoking pack-years, current smoking status, emphysema percentage, and total airway count (TAC) of the right upper lobe apical bronchus (RB1).

**Results:** PMA and PMT were lower in COPD patients, especially those with acute exacerbation, than in healthy controls. PMA and PMT were significantly associated with the severity of emphysema and the TAC of RB1 (*p* < 0.05). Patients with stable COPD, who had lower PMA and lower PMT, had significantly worse pulmonary function, poorer exercise tolerance, decreased quality of life, and worse dyspnea scores. In addition, patients with acute COPD exacerbation, who had lower PMA and lower PMT, had a higher risk of respiratory failure on admission.

**Conclusion:** CT-derived measurements of the pectoralis muscle may be helpful in detecting declines in muscle quantity and quality and predicting disease severity in patients with COPD.

## Introduction

Chronic obstructive pulmonary disease (COPD), characterized by progressive airflow limitation, remains a leading cause of death worldwide (Vogelmeier et al., 2017; Mokdad et al., 2018). Sarcopenia, a muscle-wasting syndrome, is characterized by the progressive and generalized degenerative loss of skeletal muscle mass, strength, and function, and is recognized as a critical extrapulmonary complication in COPD related to physical disability, decreased quality of life, prolonged hospitalization, and increased risk of mortality (Sepúlveda-Loyola et al., 2020). Although the decline in strength is commonly attributable to a decrease in muscle size and cross-sectional area (Hopkinson et al., 2007), muscle atrophy alone does not fully explain the muscle weakness in COPD patients, and assessing just muscle quantity is considered to be insufficient (Swallow et al., 2007; Shrikrishna et al., 2012). The consensus of the European Working Group on Sarcopenia in Older People (EWGSOP), 2018 highlighted that muscle quality is as important as muscle quantity (Cruz-Jentoft et al., 2019). Myosteatosis, which refers to excessive fat infiltration in skeletal muscles, represents low muscle quality (Aubrey et al., 2014), contributing to the declining strength and mobility (Visser et al., 2005). Previous studies have demonstrated that an increased intramuscular fat infiltration had a stronger correlation with muscle weakness and impaired mobility than muscle size in COPD, implying that myosteatosis might further explain the degree of muscle dysfunction seen in COPD (Robles et al., 2015; Shields et al., 2015). In general, early identification of muscle weakness through clinical assessment may be a promising strategy for improving individual management in patients with COPD.

Various imaging techniques are available for assessing muscle quantity, such as dual x-ray absorptiometry (DXA) (Ofenheimer et al., 2020), magnetic resonance imaging (MRI) (West et al., 2016), and computed tomography (CT). In recent years, the use of CT for research on muscle in older adults has become more common due to its ability to measure muscle quantity (e.g., cross-sectional muscle area) and muscle quality (e.g., muscle attenuation or radiodensity) (Boutin Robert et al., 2015; Lenchik and Boutin, 2018). The studies of CT-derived skeletal muscle assessment in COPD have focused largely on the limb muscles (D í az Alejandro et al., 2013; Marquis et al., 2002). On CT images, the L3 vertebral level is commonly used for abdominal muscle assessment, while the mid-thigh level is frequently used for thigh muscle assessment (Amini et al., 2019), but abdominal CT and lower extremity CT are not routine check-ups for most COPD patients. Considering that the chest CT imagining is widely used in clinical practice of COPD, we compared the densities and areas of the pectoralis muscle derived from the chest CT scan between patients with COPD and healthy controls and further explored whether the pectoralis muscle cross-sectional area (PMA) and the pectoralis muscle attenuation (PMT) were associated with the clinical characteristics of COPD patients. We hypothesized that CT-derived measurements of the pectoralis muscle could be used to detect changes in muscle quantity and quality and to predict the severity in patients with COPD.

## Patients and Methods

### Study Subjects

A total of 252 participants included in our analysis consisted 80 healthy controls and 172 patients with COPD defined by a postbronchodilator forced expiratory volume in the first second (FEV_1_) to forced vital capacity (FVC) ratio of<0.70, according to the Global Initiative for Chronic Obstructive Lung Disease (GOLD), as updated in 2017 (Vogelmeier et al., 2017). Of these patients, 85 were acute exacerbation of COPD (AECOPD) patients while 87 were stable COPD patients. Specifically, 1) The health controls included in the study were those who voluntarily went to the health examination center for a comprehensive physical examination. The inclusion criteria for the control group were subjects with normal prebronchodilator lung function who denied a history of chronic respiratory disease. 2) The stable COPD patients were enrolled from the respiratory outpatient clinics of the First Affiliated Hospital of China Medical University from July 2018 to June 2020. 3) The enrolled AECOPD patients were hospitalized in the First Affiliated Hospital of China Medical University for acute exacerbations, defined as an acute worsening of respiratory symptoms that results in additional therapy (Vogelmeier et al., 2017). The exclusion criteria comprised of populations with active lung diseases other than COPD, or cancer or long-term oral corticosteroid therapy or participating in a regular aerobic or strength exercise program, or conditions known to affect the muscle function such as musculoskeletal (e.g., knee or hip arthritis), metabolic (e.g., diabetes), or neurological disorders (e.g., stroke and Parkinson’s disease).

### Pulmonary Function and Assessments of the Modified British Medical Research Council Scale, St. George’s Respiratory Questionnaire Total Score, BODE Index, and Chronic Obstructive Pulmonary Disease Assessment Test

Spirometry and whole-body plethysmography measurement, after using a bronchodilator, was performed according to the American Thoracic Society and the European Respiratory Society guidelines (Miller et al., 2005) using a Jaeger® MasterScreen system (Jaeger®, Viasys Health care GmbH, Hochberg, Germany). The predicted spirometry and lung volume values were calculated based on the findings from Quanjer et al, (Quanjer et al., 1993). Dyspnea was measured using the Chinese version of the Modified British Medical Research Council (mMRC) dyspnea scale (Bestall et al., 1999; Cui et al., 2017), and the health status was measured using the Chinese version of the COPD Assessment Test (CAT) and St. George’s Respiratory Questionnaire (SGRQ) (Jones et al., 2009; Xu et al., 2009; Zhou et al., 2013). The BODE index, a multidimensional parameter including body-mass index (B), degree of airflow obstruction (O), functional dyspnea (D), and exercise capacity (E), was assessed according to the study proposed by Celli et al. (2004).

### Six-Minute Walk Test

According to the 2002 American Thoracic Society (ATS) guidelines (ATS Committee on Proficiency Standards for Clinical Pulmonary Function Laboratories, 2002), a closed, long, and straight 30-m indoor corridor was selected. The test method was explained to the patients before the test, and the patients were told to walk as much as possible. If they felt shortness of breath or chest pain, or dizziness, they could slow down or stop to rest. If the abovementioned symptoms worsened and were not relieved after rest, the test was stopped immediately, and the patients were supervised by the experimenter and encouraged using standardized language. After 6 min, the patients stopped when they heard an indication “time is up”. The 6MWT was measured once, and the 6-min walk distance (6MWD) was recorded in meter for analysis. The 6MWT is a standard method for assessing the exercise tolerance of COPD patients, and 6MWD<350 m indicates poor exercise tolerance (Celli et al., 2004; Cote et al., 2008).

### Computed Tomography Scan and Analysis

**FIGURE 1:**
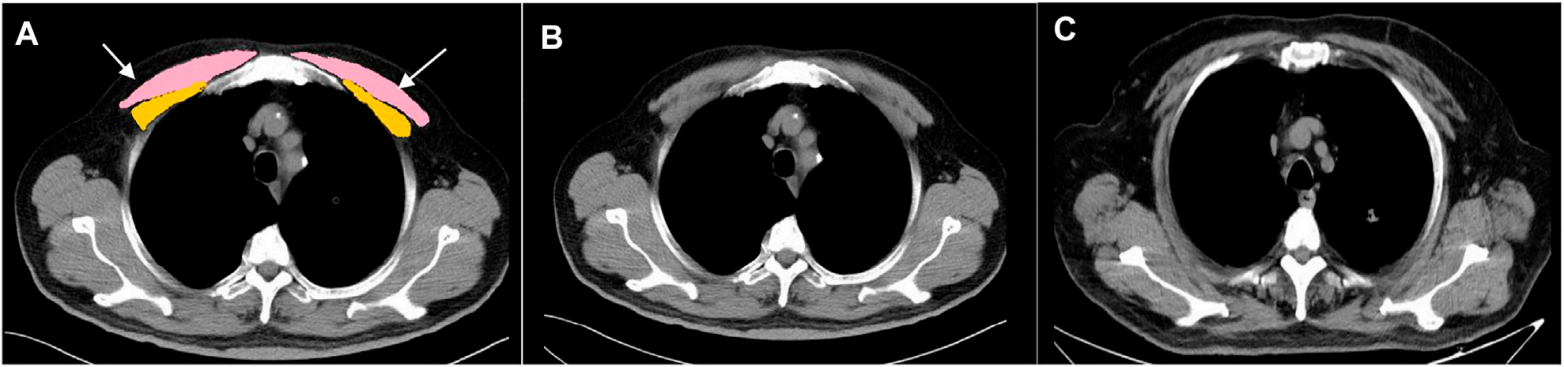
Cross-sectional muscle measurement at the level above the aortic arch. **(A)** Pectoralis muscles shaded in pink and yellow. **(B)** Healthy control (male; 65 years-old; BMI: 28.3 kg/m^2^; PMA: 31.19 cm^2^; PMT: 46.27HU). **(C)** Patient with COPD (male; 69 years-old; BMI: 28.03 kg/m^2^; PMA: 15.27 cm^2^; PMT: 29.61HU, white arrows point the adipose infiltration) PMA, pectoralis muscle cross-sectional area; PMT, pectoralis muscle attenuation; COPD, chronic obstructive pulmonary disease; BMI, body mass index; HU, Hounsfield unit.

**FIGURE 2:**
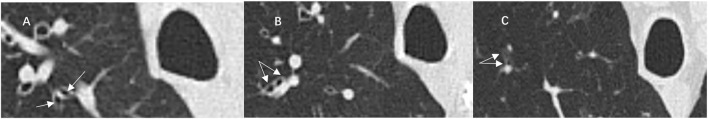
Illustrative case of computed tomography (CT) images showed distal airway branches of the right upper lobe apical bronchus (RB1). Airway branches (arrows) of the sixth **(A)**, seventh **(B),** and eighth **(C)** generations of RB1.

On the same day, patients with stable COPD underwent noncontrast chest CT scanning after being assessed for clinical traits, including pulmonary function test, 6MWT, mMRC score, SGRQ total score, and CAT score. For the AECOPD patients, their noncontrast chest CTs and arterial blood gas analysis were available as they were admitted to the hospital. Entire lung thoracic CT imaging was performed at suspended maximal inspiration after the participant coaching on a 64-slice spiral CT (Toshiba Medical Corporation, Japan). Scanning conditions: The tube ball voltage was 135 kV, the rotation time was 0.4 s, the window width was 1,600, the window position was −600, and the thickness of the reconstruction layer was 1–2 mm after continuous scanning from the bottom of the lung to the tip of the lung. On the CT images, the pectoralis muscles were examined on a single axial slice of the CT scan above the aortic arch (Figure 1A) as described previously by McDonald et al. (2014). The user visually identified the superior aspect of the aortic arch and then strolled it toward the apex of the lungs to specify the first axial image above the aortic arch because it was easily identifiable and could be consistent across a large cohort of subjects. One trained respiratory physician (XQ) performed quantitative assessments of the PMA through AutoCAD software blinded to all the subjects. The left and right pectoralis major and minor muscles were then identified on the anterior chest, and the area measurements were performed as described previously (McDonald et al., 2014) (Figure 1A). The calculated PMA reported for each scan was expressed in cm^2^ and represented the aggregate cross-sectional area of the right and left pectoralis major and minor muscles. The sum was recognized as the pectoralis muscle quantity. As described previously, the thresholds used for defining adipose tissue were from −190 Hounsfield units (HU) to −30 HU, and for the skeletal muscle, they were from −29 HU to +150 HU, which was subdivided into “normal muscle” (+30 to +150 HU) and “abnormal muscle” (−29 to +29 HU) (Mitsiopoulos et al., 1998; Aubrey et al., 2014). The PMT was measured by one rater (XQ) using a manual selection tool on a clinical workstation (IMPAX RIS). The pectoralis major and minor muscle densities were measured on both sides and in the middle and the attenuation values were recorded. Similarly, the rater made these measurements in the symmetric position. The average of all the attenuation values was recognized as the pectoralis muscle quality. Another rater (GH) measured the PMA and PMT measurements again on the same CT scans for 80 subjects to assess interobserver reproducibility. The CT DICOM data obtained were automatically determined by 3D Slicer (http://www.slicer.org/) for emphysema percentage of the whole lung below −950HU on inspiration (Gevenois et al., 1995). As described by Diaz et al. (2010), RB1 was identified, and its segmental bronchus was designated as a third airway generation (AG). RB1 was selected based on its generally perpendicular orientation to the CT scanning plane. We performed a detailed slice-by-slice examination of the CT from that segmental bronchus to identify and count its daughter branches based on Weibel’s model of airway anatomy (Weibel and Gomez, 1962). The expected number of daughter branches was calculated by the equation X = 2 ^n^, where n is the AG of interest, starting in the third AG where n was considered as 0. Based on the assumption that all the subjects would have the same number of daughter branches in the third AG, fourth AG, and fifth AG, the airway dropout was summarized as the TAC of the branches from the sixth AG to the eighth AG (Figure 2).

### Statistical Analysis

Participant characteristics were summarized as the mean (standard deviation) or proportion. The interobserver and intraobserver reliability were calculated using a two-way random model based on absolute agreement with an intraclass correlation coefficient (ICC) test. The ICC was interpreted according to the published guidelines (Shrout and Fleiss, 1979): less than 0.40, poor reliability; 0.40–0.75, fair-to-good reliability; and greater than 0.75, excellent reliability. The differences in the PMA and PMT between the healthy controls and patients with COPD were determined using covariance analysis to adjust for age, sex, and BMI. Fisher’s test was used to test the differences in muscle composition percentage between the healthy controls and patients with COPD. Pearson correlation coefficient analysis was used to compare the correlations of PMA and PMT with the baseline characteristics of all the COPD patients. Partial correlation analysis was used to compare the correlation of PMA and PMT with the emphysema percentage and TAC of RB1 adjusting for age, sex, BMI, and current smoking status, as well as to compare the correlation of PMA with PMT adjusting for age, sex, BMI, current smoking status, emphysema percentage, and TAC of RB1 in all the COPD patients. Linear regression analyses of PMA and PMT with the clinical characteristics in stable COPD patients were adjusted for age, sex, BMI, FEV_1_%pred, smoking pack-years, current smoking status, emphysema percentage, and TAC of RB1. Bivariate logistic analyses of the PMA and PMT for predicting 6MWD<350 m in stable COPD patients were adjusted for age, sex, BMI, FEV_1_%pred, smoking pack-years, current smoking status, emphysema percentage, and TAC of RB1. For AECOPD patients, bivariate logistic analyses of the PMA and the PMT for predicting respiratory failure on admission were adjusted for age, sex, BMI, current smoking status, emphysema percentage, and TAC of RB1. Sex-stratified receiver operating characteristic (ROC) curve analyses and the area under the curve (AUC) were used to determine the ability of PMA and PMT to predict 6MWD <350 m in stable COPD patients and respiratory failure in hospitalized patients with AECOPD. We determined the optimal cut-off values of PMA and PMT by calculating the Youden index (sensitivity + specificity-1) (Aggarwal and Ranganathan, 2018). All the data were analyzed using SPSS 22.0 (SPSS for Windows, version 22.0; IBM Corporation, Armonk, NY, United States). A *p* value less than 0.05 was considered statistically significant.

## Results

### Descriptive Characteristics of Study Populations

A total of 252 participants consisting of 85 AECOPD patients, 87 stable COPD patients, and 80 healthy controls, were included in the final analysis. The baseline characteristics of these participants are given in Table 1. Based on the FEV_1_% pred, after the use of a bronchodilator, the severity of airway limitation was divided into “mild-moderate” (GOLD I-II) and “severe-very severe” (GOLD III-IV). The baseline characteristics of these patients are given in Table 2.

**TABLE 1:** The baseline characteristics of the study population (*n* = 252).

	Control subjects (*n* = 80)	COPD subjects			
		Stable (*n* = 87)	p value	AE (*n* = 85)	p value
Male, %	48.8%	73.6%	0.001**	54.1%	0.297
Age, years	62.9 (7.3)	62.3 (7.8)	0.614	70.8 (10.1)	<0.001***
Height, cm	164.8 (6.5)	164.9 (7.7)	0.915	165.2 (8.8)	0.738
Weight, Kg	66.4 (10.9)	65.7 (10.0)	0.673	63.7 (14.2)	0.171
BMI, kg/m2	24.4 (3.3)	24.1 (2.9)	0.577	23.2 (4.1)	0.042*
Current smoking, %	28.7%	36.8%	0.174	52.9%	0.001**
Subject with abnormal muscle (-29HU ≤ PMT≤+29HU), %	0%	6.9%	<0.001***	22.4%	<0.001***
Emphysema percentage	——	7.8 (10.0)	——	17.6 (16.7)	——
TAC of RB1 (generations6 to 8)	——	17.8 (1.2)	——	15.8 (1.5)	——
PMA, cm2	30.4 (10.2)	27.7 (5.8)	0.034*	20.4 (7.1)	<0.001***
PMT, HU	52.6 (3.9)	42.6 (8.2)	<0.001***	35.7 (10.4)	<0.001***

Data are the mean (standard deviation) or proportion, AE, acute exacerbation; BMI, body mass index; PMA, pectoralis muscle cross-sectional area; PMT, pectoralis muscle attenuation; RB1, apical bronchus of the right upper lobe; HU, Hounsfield unit; TAC, total airway count; COPD, chronic obstructive lung disease.

Statistically significant difference compared with the control subjects (***p < 0.001, **p < 0.01, *p < 0.05).

**TABLE 2:** The baseline characteristics of patients with stable COPD.

	GOLD I–II (*n* = 68)	GOLD III–IV (*n* = 19)	p value
Smoking, pack-years	33.4 (27.0)	34.3 (29.9)	0.907
Smoker (non/ex/current), %	13.2/47.1/39.7	10.5/63.2/26.3	______
FEV_1_% pred	70.8 (13.2)	40.3 (8.0)	<0.001***
FEV_1_/FVC ratio	62.4 (7.4)	51.3 (7.0)	<0.001***
SGRQ total score	34.4 (16.8)	40.7 (14.0)	0.136
6MWD, m	390.5 (65.8)	360.3 (64.8)	0.080
CAT score	13.3 (7.8)	14.2 (4.8)	0.659
mMRC score	1.3 (1.0)	1.7 (0.9)	0.196
BODE index	1.3 (1.3)	3.4 (1.5)	<0.001***
Emphysema percentage	6.4 (8.7)	13.2 (12.8) 0.009**	<0.009***
TAC of RB1 (generations6 to 8)	17.9 (1.1)	17.5 (1.5)	0.179
PMA, cm^2^	28.1 (5.8)	26.4 (5.6)	0.035*
PMT, HU	42.9 (8.6)	41.6 (6.4)	0.264

Data are the mean (standard deviation) or proportion, AE, acute exacerbation; % pred, percentage predicted; FEV1, forced expiratory volume in 1s; FVC, forced vital capacity; BMI, body mass index; mMRC, modified Medical Research Council; CAT, chronic obstructive pulmonary disease assessment test; SGRQ, St. George’s respiratory questionnaire; BODE, body-mass index, degree of airflow obstruction, functional dyspnea, and exercise capacity; PMA, pectoralis muscle cross-sectional area; PMT, pectoralis muscle attenuation; RB1, apical bronchus of the right upper lobe; HU, Hounsfield unit; GOLD, global initiative for chronic obstructive lung disease; TAC, total airway count; 6MWD, 6-min walk distance; COPD, chronic obstructive lung diseaseStatistically significant difference between the groups (***p < 0.001, **p < 0.01, *p < 0.05).

### Reliability for Pectoralis Muscle Cross-Sectional Area and Pectoralis Muscle Attenuation Measurements

We determined the reliability level for PMA and PMT measurements in the chest CT. They were both performed by two observers and are shown in Supplementary Table S1. We assessed the intraobserver repeatability using the CT metrics of the symmetrical pectoralis muscles and they exhibited excellent ICCs (PMA: observer 1: ICC: 0.949, *p* < 0.001; observer 2: ICC: 0.955, *p* < 0.001; PMT: observer 1: ICC: 0.910, *p* < 0.001; observer 2: ICC:0.928, *p* < 0.001). Importantly, we found that the interobserver reliability for the PMA and PMT was excellent (PMA: ICC:0.993, *p* < 0.001; PMT: ICC:0.992, *p* < 0.001). These results suggested that PMA and PMT measurements using the chest CT were reliable.

### Correlations of Pectoralis Muscle Cross-Sectional Area and Pectoralis Muscle Attenuation Measurements With Participant Characteristics in Chronic Obstructive Pulmonary Disease Patients

The correlations of the PMA and the PMT with age, sex, height, weight, BMI, current smoking status, emphysema percentage, and TAC of RB1 are given in Table 3. In COPD patients, both the PMA and PMT were higher in males and younger subjects. PMA was positively associated with BMI (*r* = 0.469, *p* < 0.001), but PMT was not. The emphysema percentage was negatively correlated with PMA (*r* = −0.157, *p* = 0.045) and PMT (*r* = −0.155, *p* = 0.048), adjusting for age, sex, BMI, and current smoking status. The TAC of RB1 was positively correlated with PMA (*r* = 0.205, *p* = 0.009) and PMT (*r* = 0.237, *p* = 0.002), adjusting for age, sex, BMI, and current smoking status. In addition, PMA was also significantly associated with PMT in the COPD case subjects after adjusting for age, sex, BMI, current smoking status, emphysema percentage, and TAC of RB1 (*r* = 0.254, *p* = 0.001).

**TABLE 3:** Correlation assessment between PMA and PMT measurements and baseline characteristics in COPD.

	PMA	95% CI	*p* value	PMT	95% CI	*p* value
Sex, male	0.518	0.416 to 0.610	<0.001***	0.455	0.323 to 0.575	<0.001***
Age, years	−0.498	–0.595 to −0.397	<0.001***	−0.209	–0.350 to −0.070	0.008**
Height, cm	0.382	0.263 to 0.482	<0.001***	0.263	0.121 to 0.400	<0.001***
Weight, Kg	0.569	0.444 to 0.677	<0.001***	0.081	–0.081 to 0.227	0.292
Current smoking	0.044	–0.124 to 0.184	0.564	0.070	–0.088 to 0.202	0.362
BMI, Kg/m^2^	0.469	0.323 to 0.592	<0.001***	−0.064	–0.205 to 0.079	0.407
Adjusting for age, sex, BMI, and current smoking status						
Emphysema percentage	−0.157	–0.301 to −0.017	0.045*	−0.155	–0.289 to −0.021	0.048*
TAC of RB1	0.205	0.038 to 0.362	0.009**	0.237	0.061 to 0.386	0.002**
Adjusting for age, sex, BMI, current smoking status, emphysema percentage and TAC of RB1						
PMT, HU	0.254	0.114 to 0.385	0.001**			

BMI, body mass index; RB1, apical bronchus of right upper lobe; PMA, pectoralis muscle cross-sectional area; PMT, pectoralis muscle attenuation; TAC, total airway count; COPD, chronic obstructive lung disease; CI, confidence interval. ***p < 0.001, **p < 0.01, *p < 0.05.

### Pectoralis Muscle Cross-Sectional Area and Pectoralis Muscle Attenuation in Healthy Controls and Patients With Chronic Obstructive Pulmonary Disease Patients

**FIGURE 3:**
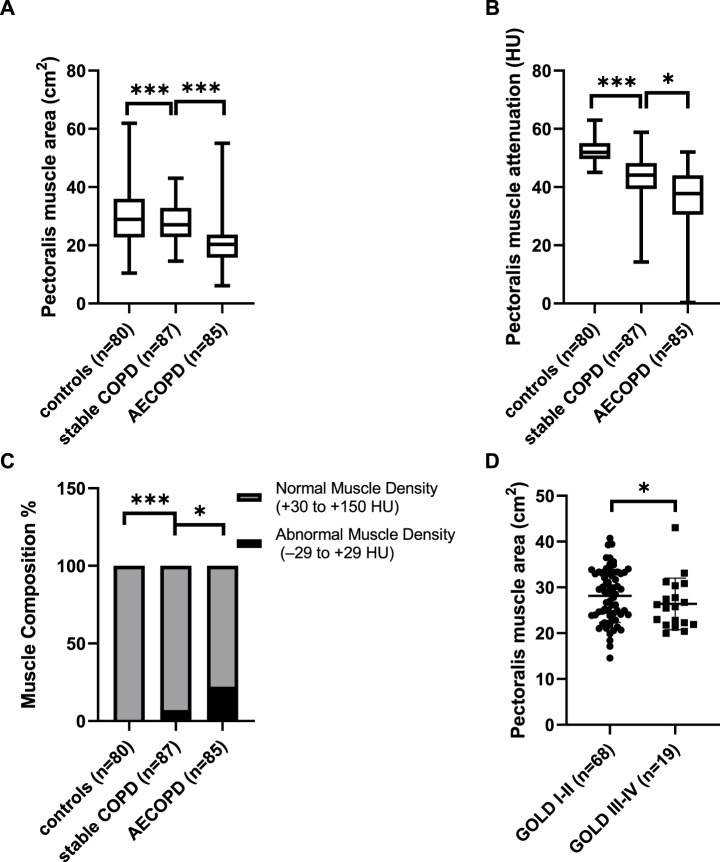
The differences of the pectoralis muscle cross-sectional area (PMA), pectoralis muscle attenuation (PMT), and abnormal muscle proportion between the patients with chronic obstructive pulmonary disease (COPD) and healthy controls. **(A)** Difference in PMA between the patients with COPD and healthy controls adjusting for age, sex, and body-mass index (BMI), **(B)** difference in PMT between the patients with COPD and healthy controls adjusting for age, sex, and BMI; **(C)** difference in the proportion of subjects with abnormal muscle (−29 HU ≤ PMT≤+29 HU) between patients with COPD and healthy controls; **(D)** difference in PMA between the patients with global initiative for chronic obstructive lung disease (GOLD) I-II and patients with GOLD III-IV adjusting for age, sex, and BMI. *p < 0.05; ***p < 0.001. AE, acute exacerbation; HU, Hounsfield unit.

Representative pictures of PMT and PMA measurements in the healthy controls and patients with COPD are shown in Figure 1. We found that PMA and PMT were lower in the COPD patients, especially those with acute exacerbation, than in the healthy controls after adjusting for age, sex, BMI, and current smoking status (Figures 3A,B). The percentage of subjects with “abnormal muscle” was significantly higher in COPD patients, especially those with acute exacerbation, than in the healthy controls (Figure 3C). In addition, the PMA of patients with GOLD stage I-II was significantly higher than that of patients with GOLD stage III-IV after adjusting for age, sex, and BMI (Figure 3D). However, there was no statistical significance in PMT between the patients with GOLD stage I-II and those with GOLD stage III-IV after adjusting for age, sex, and BMI.

### Relationship of Pectoralis Muscle Cross-Sectional Area and Pectoralis Muscle Attenuation With Clinical Characteristics in Stable Chronic Obstructive Pulmonary Disease Patients

As shown in Table 4, we established the linear regression models of PMA and PMT with clinical characteristics in stable COPD patients. The PMA and PMT were statistically correlated with FEV_1_%pred and FEV_1_/FVC, adjusting for age, sex, BMI, smoking pack-years, current smoking status, emphysema percentage, and TAC of RB1. They were both negatively correlated with the SGRQ total score, mMRC score, and BODE index and positively correlated with 6MWD adjusting for age, sex, BMI, smoking pack-years, FEV_1_%pred, current smoking status, emphysema percentage, and TAC of RB1. These results suggested that patients with stable COPD who had lower PMA and lower PMT had significantly worse pulmonary function, poorer exercise tolerance, worse prognosis, and decreased quality of life. For example, an average increase of 1 cm^2^ in PMA was significantly associated with an increase of 1.445% predicted FEV_1_ (95% CI, 0.646 to 2.243; *p =* 0.001) and an increase of 0.638 FEV_1_/FVC ratio (95% CI, 0.267 to 1.009; *p* = 0.001). Similarly, an average of one HU increase in the PMT was significantly associated with an increase of 0.541% predicted FEV_1_ (95% CI, 0.025 to 1.057; *p* = 0.040) and an increase of 0.358 FEV_1_/FVC ratio (95% CI, 0.124 to 0.591; *p* = 0.003). In particular, an average of 1 cm^2^ increase in the PMA and an average of one HU increase in PMT were associated with 4.697 m (95% CI, 1.514 to 7.881; *p* = 0.004) and 2.290 m (95% CI, 0.384 to 4.195; *p* = 0.019) increases in 6MWD, respectively. Given that, low BMI and high BMI may both influence the result of 6MWD, we categorized BMI into low, normal, overweight, and obese. As shown in Table 5, we did not find significant differences in 6MWD among the four BMI categories, which might result from a small sample size in both the low and obese groups. To eliminate the effect of BMI classification on 6MWD, we performed a new linear regression analysis adjusted for age, sex, BMI classification, smoking pack-years, FEV_1_%pred, current smoking status, emphysema percentage, and TAC of RB1 (Table 6). We found that a 1 cm^2^ increase in the PMA and a one HU increase in the PMT were associated with 4.926 m (95% CI, 1.866 to 7.987; *p* = 0.002) and 2.204 m (95% CI, 0.317 to 4.091; *p* = 0.023) increases in 6MWD, respectively. In addition, there was no linear regression relationship between PMA and PMT after adjusting for age, sex, BMI, smoking pack-years, FEV_1_%pred, current smoking status, emphysema percentage, and TAC of RB1. However, we found a linear regression relationship between PMA and PMT (*β* = 0.172, 95% CI, 0.037 to 0.308; *p* = 0.013) when FEV_1_%pred was not controlled, which suggested that the linear correlation between PMA and PMT was dependent on FEV_1_%pred.

**TABLE 4:** The regression analyses between PMA and PMT with clinical characteristics in patients with stable COPD.

Predictor	Mean difference per cm^2^ increment in the pectoralis muscle cross-sectional area	95% CI	*p* value	Mean difference per HU Increment in pectoralis muscle attenuation	95% CI	*p* value
Adjusting for age, sex, BMI, smoking pack-years, current smoking status, emphysema percentage, and TAC of RB1						
FEV_1_, % pred	**1.445**	**0.646 to 2.243**	0.001**	**0.541**	**0.025 to 1.057**	0.040*
FVC, % pred	**1.013**	**0.081 to 1.945**	0.034*	**0.395**	**–0.218 to 1.009**	**0.203**
FEV_1_/FVC	**0.638**	**0.267 to 1.009**	0.001**	**0.358**	**0.124 to 0.591**	0.003**
Adjusting for age, sex, BMI, FEV_1_%pred, smoking pack-years, current smoking status, emphysema percentage, and TAC of RB1						
SGRQ total score	**−1.033**	**–1.782 to −0.284**	0.008**	**−0.598**	**–1.040 to −0.157**	0.009*
6MWD	**4.697**	**1.514 to 7.881**	0.004**	**2.290**	**0.384 to 4.195**	0.019*
BODE	**−0.086**	**–0.145 to −0.027**	0.005**	**−0.042**	**–0.078 to −0.007**	0.019*
mMRC	**−0.053**	**–0.102 to −0.004**	0.034*	**−0.039**	**–0.067 to −0.010**	0.009**
CAT	**0.083**	**–0.294 to 0.459**	**0.664**	**−0.133**	**–0.353 to 0.087**	**0.232**
PMA, cm^2^				**0.128**	**–0.003 to 0.259**	**0.055**
Adjusting for age, sex, BMI, smoking pack-years, current smoking status, emphysema percentage, and TAC of RB1						
PMA, cm^2^				**0.172**	**0.037 to 0.308**	0.013*

% pred, percentage predicted; FEV1, forced expiratory volume in 1s; FVC, forced vital capacity; BMI, body mass index; mMRC, modified Medical Research Council; CAT, chronic obstructive pulmonary disease assessment test; SGRQ, St. George’s respiratory questionnaire; BODE, body-mass index, degree of airflow obstruction, functional dyspnea, and exercise capacity; PMA, pectoralis muscle cross-sectional area; PMT, pectoralis muscle attenuation; RB1, apical bronchus of the right upper lobe; TAC, total airway count; 6MWD, 6-min walk distance; COPD, chronic obstructive lung disease; CI, confidence interval. **p < 0.01, *p < 0.05.

**TABLE 5:** The baseline characteristics of patients with stable COPD when the BMI classification was carried out.

	Low (<18.5 kg/m^2^)	Normal (18.5-24.9 kg/m^2^)	Overweight (25-29.9 kg/m^2^)	Obese (≥30kg/m^2^)
Number	1	52	31	3
6MWD, m	282.00	388.70 (66.64)	384.39 (65.74)	329.67 (33.86)
PMA, cm^2^	25.47	26.00 (5.07)	30.58 (5.30) *	29.43 (11.78)
PMT, HU	41.49	43.19 (8.27)	42.19 (8.47)	38.36 (4.51)

Data are mean (standard deviation), COPD , chronic obstructive lung disease; BMI, body mass index; PMA, pectoralis muscle cross-sectional area; PMT, pectoralis muscle attenuation; 6MWD, 6-minute-walk distance.*p < 0.05.

**TABLE 6:** The regression analyses between the PMA and PMT measurements and 6MWD in patients with stable COPD adjusting for age, sex, BMI classification, smoking pack-years, FEV1%pred, current smoking status, emphysema percentage, and TAC of RB1.

Predictor	Mean Difference per cm^2^ Increment in pectoralis muscle cross-sectional area	95% CI	*P*-value	Mean Difference per HU Increment in pectoralis muscle attenuation	95% CI	*P*-value
6MWD	4.926	1.866 to 7.987	0.002**	2.204	0.317 to 4.091	0.023*

BMI, body mass index; 6MWD, 6-minute-walk distance; COPD, chronic obstructive lung disease; PMA, pectoralis muscle cross-sectional area; PMT, pectoralis muscle attenuation; RB1, apical bronchus of right upper lobe; TAC, total airway count; % pred, percentage predicted; FEV1, forced expiratory volume in 1s; CI, confidence interval. **p < 0.01, *p < 0.05.

### Lower Pectoralis Muscle Cross-Sectional Area and Lower Pectoralis Muscle Attenuation Predicted the Risk of Respiratory Failure in Acute Exacerbation of Chronic Obstructive Pulmonary Disease Patients, and Lower Pectoralis Muscle Cross-Sectional Area Predicted the Risk of Exercise Intolerance in Stable Chronic Obstructive Pulmonary Disease Patients

Among the stable COPD patients, 27 had poor exercise tolerance (6MWD<350 m). Bivariate logistic analysis (Table 7) showed that the risk of exercise intolerance increased by 26.3% with a decrease of 1 cm^2^ in PMA (OR = 1.263, 95% CI, 1.072 to 1.487; *p* = 0.005), while there was no significance observed in PMT after adjusting for age, sex, BMI, smoking pack-years, FEV_1_%pred, current smoking status, emphysema percentage, and TAC of RB1. In the AECOPD group, 29 patients were diagnosed with respiratory failure according to the arterial blood gas analysis (PaO2<60 mmHg) without oxygen therapy on admission. The risk of respiratory failure increased by 14.1% (OR = 1.141, 95% CI, 1.002 to 1.299; *p* = 0.047) with a decrease of 1 cm^2^ in PMA as well as increased by 17.8% (OR = 1.178, 95% CI, 1.088 to 1.276; *p* < 0.001) with a decrease of one HU in PMT after adjusting for age, sex, BMI, current smoking status, emphysema percentage, and TAC of RB1.

**TABLE 7:** Bivariate logistic analyses of the PMA and the PMT for predicting 6MWD< 350 m and respiratory failure.

	PMA			PMT		
	Odds ratio	95% CI	p value	Odds ratio	95% CI	p value
6MWD<350 m (stable COPD)	1.263	1.072 to 1.487	0.005**	1.066	0.989 to 1.149	0.093
Respiratory failure (AECOPD)	1.141	1.002 to 1.299	0.047*	1.178	1.088 to 1.276	< 0.001***

PMA, pectoralis muscle cross-sectional area; PMT, pectoralis muscle attenuation; 6MWD, 6-minute-walk distance; AE, acute exacerbation; 6MWD, 6-minute walk distance; COPD, chronic obstructive lung disease. ***p < 0.001, *p < 0.05.

### The Potential Values of Pectoralis Muscle Cross-Sectional Area and Pectoralis Muscle Attenuation in the Prediction of Exercise Intolerance in Stable Chronic Obstructive Pulmonary Disease Subjects and Respiratory Failure in Acute Exacerbation of Chronic Obstructive Pulmonary Disease Subjects When Stratified by Sex

**FIGURE 4:**
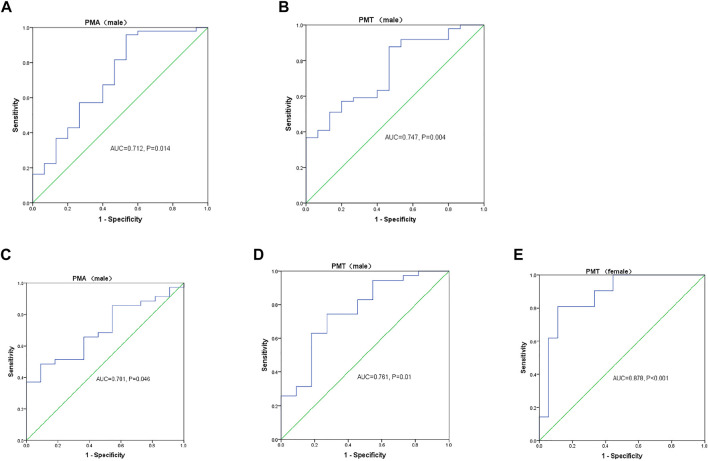
Sex-stratified receiver operating characteristic (ROC) curve analyses of the pectoralis muscle cross-sectional area (PMA) and pectoralis muscle attenuation (PMT) measurements for predicting **(A,B)** 6-min walk distance (6MWD) < 350 m in patients with stable chronic obstructive lung disease (COPD) and **(C–E)** respiratory failure in patients with acute exacerbation of COPD. AUC, area under the curve.

Due to the effect of sex on PMA and PMT, we performed the sex-stratified ROC analyses. In the stable group, 15 male and 12 female patients had poor exercise tolerance (6MWD<350 m). As shown in Figure 4, the AUC values for PMA (Figure 4A) and PMT (Figure 4B) were 0.712 and 0.747, respectively. In addition, 23.95 cm^2^ and 41.05 HU was recognized as the optimal threshold values for predicting exercise intolerance in the male patients. However, the AUC values for PMA and PMT were not statistically significant in the female patients. Of the 85 patients with AECOPD, 11 male patients and 18 female patients were diagnosed with respiratory failure. The AUC values for PMA (Figure 4C) and PMT (Figure 4D) were 0.701 and 0.761, respectively. In addition, 24.43 cm^2^ and 40.75 HU was recognized as the optimal threshold values for predicting respiratory failure in the male patients. The AUC value for PMT (Figure 4E) was 0.878, and 33.58 HU were recognized as the optimal threshold values for predicting respiratory failure in the female patients, while the AUC value for PMA was not statistically significant.

## Discussion

The present study evaluated the clinical value of CT-derived PMA and PMT in the COPD patients at different stages and investigated the relationship between PMA and PMT. We demonstrated that PMA and PMT were lower in the COPD patients, especially those with acute exacerbation, than in the healthy controls. PMA and PMT were both associated with lung function, exercise capacity, quality of life, and dyspnea score. These associations were stronger in PMA than in PMT, suggesting that PMA was more sensitive to COPD severity than PMT. We also found that PMA was positively correlated with PMT and this correlation was dependent on FEV_1_%pred, but not emphysema. In addition, we confirmed that a lower PMA could effectively predict exercise intolerance and respiratory failure, while a lower PMT could only predict respiratory failure in patients with COPD.

The usage of the term “sarcopenia” in the medical literature is inconsistent. For example, the ageing literature reserves “sarcopenia” for low muscle mass and low physical function and considers myosteatosis as a related but distinct entity (Bianchi et al., 2017; Schaap et al., 2018; Tang et al., 2018). In contrast, many cancer journals use the term “sarcopenia” for low muscle mass and occasionally consider myosteatosis as a component of sarcopenia rather than a distinct entity (Kumar et al., 2016; Lenchik et al, 2019; Rier et al., 2017). There is no established gold standard to quantify myosteatosis (Correa-de-Araujo et al., 2017; Correa-de-Araujo et al., 2020). Myosteatosis was usually estimated by the attenuation of skeletal muscle on the CT scans (Martin et al., 2013; Fujiwara et al., 2015), and the values of <41 HU or <33 HU in the total abdominal musculature were used as an indicator for myosteatosis in both men and women, depending on BMI (Cooper et al., 2015; Henderson et al., 2016). Although three suggested constituents that could cause a marked decrease in attenuation in the muscle: lipid, glycogen, and water (D é riaz et al., 2001), glycogen and water have been eliminated as possibilities due to theoretical considerations (D é riaz et al., 2001). Goodpaster et al. (1985); Larson-MeyerEnette et al. (2006) also confirmed that reduced muscle attenuation was directly associated with lipid accumulation. The biochemical and histological analysis also supports that the mean attenuation/density of muscle measured by CT reflects the lipid content (Kelley et al., 1991). Tachi et al. (Tachi et al., 2018) confirmed that myosteatosis is associated with the skeletal muscle volume loss and that skeletal muscle attenuation has utility in predicting skeletal muscle volume loss in patients with chronic liver disease. Few studies have explored the association between muscle quantity and muscle quality in COPD. Our study found that PMA was positively associated with PMT in the COPD patients independent of emphysema severity and TAC of RB1. There was a linear regression relationship between PMA and PMT dependent on FEV_1_%pred, which suggested a potential link between the muscle mass loss and intramuscular fat infiltration when there was airflow limitation rather than emphysema. Previous studies have indicated that elevated levels of toxic lipid metabolites and adipose-derived cytokines could impair the signaling and function of muscle cells, resulting in skeletal muscle wasting (Dyck et al., 2006; Zoico et al., 2010; Nicholson et al., 2018; Mannelli et al., 2020). However, the exact mechanism of myosteatosis and molecular pathways, how it affects COPD patients’ skeletal muscle function and how it may cause muscle volume loss, remain unclear and need further investigation.

Sarcopenia and myosteatosis have been documented as increasing as a person’s age (Evans and Campbell, 1993; Hamrick et al., 2016), which is consistent with our findings that PMA and PMT were lower in older patients with COPD. In addition, although BMI have been conventionally used to assess muscle wasting in patients with COPD (Celli et al., 2004; Schols et al., 2005), our present study found that BMI was positively correlated with PMA, but not PMT. Thus, we thought that BMI might not accurately represent muscle wasting, while muscle quantity and quality measurements would constitute a more comprehensive assessment of muscle wasting than BMI in COPD. On the other hand, we also found that PMA and PMT were lower in COPD patients, especially those with acute exacerbation, than in the healthy controls. For the stable COPD patients, the PMA of patients with GOLD stage I-II was significantly higher than that of patients with GOLD stage III-IV, supporting a previous study that found that sarcopenia was significantly higher in patients with GOLD stage III-IV than in those with GOLD stage I–II (Sepúlveda-Loyola et al., 2020). However, there was no statistical significance in the PMT between the patients with GOLD stage I-II and those with GOLD stage III-IV, adjusting for age, sex, and BMI. It could be considered that the PMA was more sensitive to COPD severity than the PMT. Moreover, we further explore the correlation between PMA and PMT measurements and emphysema severity and TAC, whose reduction predicted more rapid lung function decline (Kirby et al., 2018). We found that PMA and PMT were negatively associated with the emphysema percentage and positively associated with the TAC of RB1. These findings supported our hypothesis that CT-derived PMA and PMT might be clinically significant to detect declines in muscle quantity and quality and predict COPD severity.

Donovan et al. (2021) previously quantified the pectoralis and paraspinal muscle densities and areas on the chest CT from mild-to-very severe COPD patients and observed independent associations with the disease severity, exacerbations, impaired health status, and exercise capacity. Consistently, our present study found that PMA and PMT were negatively associated with the SGRQ total score, mMRC score, and BODE index, and positively associated with FEV_1_%pred, FEV_1_/FVC, and 6MWD, independent of emphysema severity and TAC of RB1, suggesting that those patients with lower PMA and lower PMT had reduced pulmonary function, poor exercise tolerance, decreased quality of life, and worse dyspnea score. Moreover, we found that a lower PMA could predict the risk of exercise intolerance in the stable COPD subjects. Although the prediction conducted by logistic regression analysis was not observed in PMT, we found that 41.05 HU was the optimal threshold value for exercise intolerance in male patients through ROC analysis. In addition, male COPD patients with PMA less than 23.95 cm^2^ were considered to be at a risk for exercise intolerance. Perrot et al. (2020) found a high prevalence of sarcopenia in patients hospitalized during an acute exacerbation of COPD on admission (48%), and respiratory muscle strength measured by MIP and MEP was lower in the sarcopenic patients. Muscle wasting could also affect the respiratory muscles, and muscle wasting could be both a cause and a consequence of respiratory problems (Lim et al., 2011), as demonstrated by the correlation between PMA and PMT measurements and lung function in our stable COPD patients. Moreover, lower PMA and lower PMT predicted the risk of respiratory failure in the AECOPD patients, and those hospitalized male patients with PMA less than 24.43 cm^2^ or PMT less than 40.75 HU, while those female patients with PMT less than 33.58 HU were more likely to develop respiratory failure on admission. As proposed by Cooper et al. (Henderson et al., 2016) the value of <41 HU or <33 HU in the total abdominal musculature was used to indicate myosteatosis in both men and women, depending on the BMI. Therefore, we supposed that patients with myosteatosis were prone to exercise intolerance and respiratory failure. Taken together, PMA and PMT might be promising tools for assessing the COPD manifestation and prognosis.

However, there are limitations to this investigation. First, we assessed the pectoralis muscle area at the level above the aortic arch, which cannot represent the entire pectoral muscle area, and the cross-section was not guaranteed to be completely identical in all the participants. Second, the sample was limited to the northern Chinese population. The clinical application of CT assessment of the pectoralis, whether it would be generalizable to COPD patients in other countries, needs further comprehensive and in-depth analysis. The healthy controls from the medical examination center did not perform postbronchodilator spirometry, which could not rule out conditions such as asthma. In addition, stable COPD patients were continuously enrolled from the outpatient settings for certain periods of time, so there were sex differences between the stable COPD patients and healthy controls, and AECOPD patients. To minimize the impact of section bias, subsequent statistical analyses are adjusted for confounding factors, such as age, sex, and BMI. Third, this retrospective study limited our ability to comprehensively collect physical activity (e.g., gait speed), glucocorticoids medication, and detailed smoking status (e.g., nonsmoker/ex-smoker). Fourth, due to the small sample size, the established cut-off values for PMA and PMT did not take BMI or age into account. Fifth, it is true that a repeat 6MWT is recommended to balance the underperformance on the initial test in those lacking familiarity and underperformance on the subsequent 6MWT from fatigue. Due to the limited outpatient setting and unpleasant feelings after the 6MWT, the patients completed the 6MWT only once, which may lead to inaccurate results. Finally, respiratory failure was also affected by multiple factors, such as infection severity and complications of heart disease, which were not documented in our study.

In conclusion, chest CT-derived PMA and PMT were lower in the COPD patients than in the healthy controls. They were both associated with disease severity, quality of life, and exercise capacity. Additionally, AECOPD patients, who had lower PMA and lower PMT, had a higher risk of respiratory failure on admission. Our results demonstrated that chest CT scan, a widely used examination in COPD, could be a valuable and feasible tool for assessing muscle quantity and quality, which will be helpful in evaluating sarcopenia and myosteatosis. Using the chest CT to detect changes in muscle quantity and quality could facilitate the development of individualized interventions (e.g., refined nutritional, pulmonary rehabilitation, inspiratory muscle training, and pharmaceutical), as well as estimate the prognosis of COPD exacerbation, which may result in meaningful improvements in the outcomes and quality of life for COPD patients. Nevertheless, a longitudinal study of the long-time effects of the pectoralis measured by chest CT on lung function, exacerbation, malnutrition, and exercise tolerance is needed.

## Data Availability

The original contributions presented in the study are included in the article/Supplementary Material, further inquiries can be directed to the corresponding authors.
