# Supplementary material for: CT Attenuation and Cross-Sectional Area of the Pectoralis Are Associated With Clinical Characteristics in Chronic Obstructive Pulmonary Disease Patients
Source: Front Physiol. 2022 Jun 3;13:833796. doi: 10.3389/fphys.2022.833796 (PMC9205603; doi:10.3389/fphys.2022.833796)
Supplement: Supplementary file 1 [file Table1.DOCX]

**Table 1. Reliability and repeatability results of PMA and PMT measurements performed by different observers**

| Parameter | **Observer 1** |  | **Observer 2** |
| --- | --- | --- | --- |
| **Intra-observer** |  |  |  |
| **PMA** |  |  |  |
| ICC (95%CI) | 0.949(0.934-0.960) |  | 0.955(0.943-0.965) |
| *P* value | <0.001*** |  | <0.001*** |
| **PMT** |  |  |  |
| ICC (95%CI) | 0.910(0.885-0.930) |  | 0.928(0.908-0.944) |
| *P* value | <0.001*** |  | <0.001*** |
| **Interobserver** |  |  |  |
| **PMA** |  |  |  |
| ICC (95%CI) |  | 0.993(0.992-0.995) |  |
| *P* value |  | <0.001*** |  |
| **PMT** |  |  |  |
| ICC (95%CI) |  | 0.992(0.990-0.994) |  |
| *P* value |  | <0.001*** |  |

Abbreviations: ICC= intraclass correlation coefficient; PMA=pectoralis muscle cross-sectional area; PMT= pectoralis muscle attenuation; CI=confidence interval. ****P*<0.001
